# Proceedings of the Chicago Gynæcological Society

**Published:** 1879-08

**Authors:** 


### Society Reports.

### ARTICLE IX.

PROCEEDINGS OF THE CHICAGO GYNÆCOLOGICAL SOCIETY. Eighth Meeting, May 23, 1879.

DR. T. D. FITCH in the chair.

DR. EARLE read a paper on tubercular meningitis (published in July number of JOURNAL AND EXAMINER).

### DISCUSSION.

DR. MILLER asked if every case of acute meningitis might not be associated with tubercle. He admitted the great difficulty of diagnosis. It was certain, however, that he had witnessed recoveries in cases of meningitis in which there were evidences of tubercle. In the treatment of these cases the speaker believed in shaving the scalp, applying blisters and stimulating all the excretory channels. It was questionable whether bromides were of service.

DR. MERRIMAN mentioned a case of supposed tubercular meningitis, which recovered. He, too, agreed as to the extreme difficulty of diagnosis. Theoretically, he could not understand why recovery from tubercular meningitis should be so infrequent.

DR. BYFORD objected to the name, tubercular meningitis. He thought usually the meningitis preceded the formation of tubercle. Often, however, those two kinds of cases merged into each other, and the diagnosis is thus made extremely difficult. Death in these cases is caused by compression of the brain. Concerning the treatment, he had seen several cases recover under the effects of ergot and quinine. In answer to Dr. Miller, the speaker said he would not administer full doses of quinine when there were evidences of cerebral congestion of a considerable degree.

DR. NELSON accepted Dr. Byford's views as to the real nature of these cases. He alluded to the necessity of an energetic line of treatment: cold applied to the head, quinine in every case of high temperature, ergot, bromides, and digitalis and belladonna to support the heart's action. He adopted the use of blisters, but on account of the disfigurement, which is shocking to the friends in case of death, he used small blisters, on the nape of the neck, behind the ears, and in other concealed places.

DR. SAWYER alluded to the fact that an unusual number of cases had come under the observation of the essayist. He had been led to regard the affection as a less common one. In both the cases which he had seen, the evidences of tuberculosis of other parts of the body overshadowed at first the cerebral manifestations. In one of the cases there were tubercles in the choroid.

DR. DUDLEY spoke of the theoretical use of alcohol in meningitis.

DR. FITCH remarked that the great, distinguishing feature of tubercular meningitis was its slow progress, and its indefinite character. It seems sub-acute from the outset. He thought the majority of the cases reported in the essay were like those of simple meningitis.

---

CHICAGO MEDICAL COLLEGE.—The following appointments have been recently made to fill vacancies in the faculty of this college: Edward W. Jenks, M.D., LL.D., formerly of the Detroit Medical College, has been appointed to the chair of Medical and Surgical Diseases of Women and of Clinical Gynæcology, and will soon take up residence in this city. Lester Curtis, A.M., M.D., formerly assistant or adjunct to the Chair of Physiology and Histology, has been appointed Professor of Histology. H. Gradle, M.D., occupies the Chair of Physiology temporarily, as Lecturer in that department.

